# Disruption of Higher Order DNA Structures in Friedreich’s Ataxia (GAA)_n_ Repeats by PNA or LNA Targeting

**DOI:** 10.1371/journal.pone.0165788

**Published:** 2016-11-15

**Authors:** Helen Bergquist, Cristina S. J. Rocha, Rubén Álvarez-Asencio, Chi-Hung Nguyen, Mark. W. Rutland, C. I. Edvard Smith, Liam Good, Peter E. Nielsen, Rula Zain

**Affiliations:** 1 Department of Medical Biochemistry and Microbiology, Microbiology-Immunology, Uppsala University, Uppsala, Sweden; 2 Department of Laboratory Medicine, Clinical Research Center, Karolinska Institutet, SE-141 86, Huddinge, Sweden; 3 KTH Royal Institute of Technology, School of Chemical Science and Engineering, Department of Chemistry, Stockholm, Sweden; 4 Laboratoire de Pharmacochimie, Institut Curie, PSL Research University, UMR 9187 – U 1196 CNRS-Institut Curie, INSERM, Centre Universitaire, Orsay, France; 5 Department of Pathology and Infectious Diseases, Royal Veterinary College, University of London, United Kingdom; 6 Department of Cellular and Molecular Medicine, Faculty of Health and Medical Sciences, University of Copenhagen, The Panum Institute, Copenhagen, Denmark; 7 Department of Clinical Genetics, Centre for Rare Diseases, Karolinska University Hospital, SE-171 76, Stockholm, Sweden; University of Queensland, AUSTRALIA

## Abstract

Expansion of (GAA)_n_ repeats in the first intron of the *Frataxin* gene is associated with reduced mRNA and protein levels and the development of Friedreich’s ataxia. (GAA)_n_ expansions form non-canonical structures, including intramolecular triplex (H-DNA), and R-loops and are associated with epigenetic modifications. With the aim of interfering with higher order H-DNA (like) DNA structures within pathological (GAA)_n_ expansions, we examined sequence-specific interaction of peptide nucleic acid (PNA) with (GAA)_n_ repeats of different lengths (short: n=9, medium: n=75 or long: n=115) by chemical probing of triple helical and single stranded regions. We found that a triplex structure (H-DNA) forms at GAA repeats of different lengths; however, single stranded regions were not detected within the medium size pathological repeat, suggesting the presence of a more complex structure. Furthermore, (GAA)_4_-PNA binding of the repeat abolished all detectable triplex DNA structures, whereas (CTT)_5_-PNA did not. We present evidence that (GAA)_4_-PNA can invade the DNA at the repeat region by binding the DNA CTT strand, thereby preventing non-canonical-DNA formation, and that triplex invasion complexes by (CTT)_5_-PNA form at the GAA repeats. Locked nucleic acid (LNA) oligonucleotides also inhibited triplex formation at GAA repeat expansions, and atomic force microscopy analysis showed significant relaxation of plasmid morphology in the presence of GAA-LNA. Thus, by inhibiting disease related higher order DNA structures in the *Frataxin* gene, such PNA and LNA oligomers may have potential for discovery of drugs aiming at recovering *Frataxin* expression.

## Introduction

Friedreich’s ataxia (FRDA) is the most common inherited autosomal recessive ataxia, and in >96% of cases the disease is correlated by expansion of (GAA)_n_ repeats in the first intron of the *Frataxin* gene (*FXN*) [1,2]. Disease-associated expanded alleles consist of approximately 70 to more than 1000 repeats [1–3]. The (GAA)_n_ expansions result in a substantial reduction in *Frataxin* mRNA and protein levels [4], and non-symptomatic carriers (heterozygous for the expanded allele) show ~50% reduction [1,3–5]. Frataxin deficiency causes excessive free radical production, dysfunction of Fe-S center containing enzymes, and progressive iron accumulation in mitochondria [6]. Several studies have focused on increasing the level of *Frataxin* through targeting the epigenetic regulation of gene expression. Unfortunately, there are no FRDA therapeutics available, only symptomatic management strategies.

**Fig 1:**
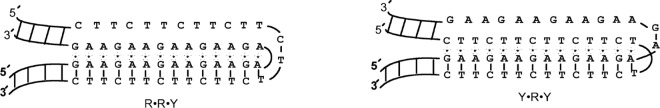
Purine and pyrimidine H-DNA motifs formed at (GAA)_n_ repeats.

Expanded (GAA)_n_ repeats readily form non-canonical (non-B-DNA) structures including intramolecular triplex structures (H-DNA) (Fig 1), and R-loops, which have also been proposed to be of importance in other triplex-repeat disorders [7]. Several models have been proposed for alternative structures formed at expanded (GAA)_n_ repeats [8–12]. Formation of a higher order structure named “sticky DNA” has been reported in (GAA)_n_ containing plasmids and the structure was analyzed using electron microscopy [8,13–16]. It is believed that the structural properties of (GAA)_n_ repeats that lead to the formation of higher order structures also affect the genomic stability of the repeat length as well as the expression of *Frataxin* [17–19]. Long (GAA)_n_ repeats stall replication in *Saccharomyces cerevisiae* [20] and inhibit transcription both *in vitro* [5,21] and in mammalian cells [13,22]. The observed effects on DNA replication and transcription are dependent on the length and orientation of the (GAA)_n_ repeats, which correlate with the predisposition of these repeats to form well-defined secondary/tertiary DNA structures [7,23]. Finally, the expanded repeats are associated with silenced chromatin via DNA methylation and histone trimethylation and deacetylation in the adjacent regions [24,25].

An understanding of the DNA structural properties and chromatin modifications associated with (GAA)_n_ repeats raises possibilities to reverse *Frataxin* silencing. For example, histone deacetylase inhibitors have been used to increase *Frataxin* mRNA in FRDA mouse models and in patient cell lines [26–29]. Also, sequence-specific polyamides and low molecular weight minor groove binders enhance *Frataxin* expression [30,31]. Therefore, it is particularly interesting to develop specific (GAA)_n_ repeat targeting molecules to elucidate the possible pathological structures formed at the *Frataxin locus*, which could facilitate the development of new therapeutic strategies.

The GAA expanded repeats in FRDA consist of large polypurine.polypyrimidine (R.Y) regions. In principle, these stretches can be specifically targeted by triplex forming oligonucleotides (TFOs), which bind in the major groove of the dsDNA. This, so-called anti-gene strategy, can be used to modulate transcription at specific *loci* [32–34] and to induce recombination [35,36] and repair [37–39]. For example, to enhance TFO binding of DNA under physiological conditions, modified nucleotides, such as morpholinophosphoroamidates (PMO), locked (LNA) or peptide nucleic acids (PNA) can be exploited [40].

PNAs are DNA mimics having a peptide like backbone [41]. PNA binds to sequence complementary DNA or RNA with high affinity and sequence specificity. More interestingly, PNA is able to invade dsDNA through binding of the purine (R) strand leaving the pyrimidine (Y) strand displaced. PNA was originally designed to bind dsDNA to form a triplex structure; however, it was soon discovered that an invasion mechanism is involved and several other PNA-DNA complexes can also be formed [41]. Binding of short homopyrimidine PNA to dsDNA leads mainly to formation of a triplex-invasion structure, of very high stability [42]. Formation of a triplex-invasion complex is slow and negatively affected by DNA duplex stabilizing conditions, such as physiological salt concentrations [43]. Formation of triplex-invasion structures requires two PNA molecules for Watson-Crick and Hoogsteen binding, respectively, of the target. To increase the rate of formation of a triplex-invasion complex, bis-PNAs, where two PNA molecules are linked together, have been developed [44]. Duplex invasion complexes have been reported for homopurine PNAs [45] and also for backbone modified, high affinity PNAs [46] (*e*.*g*. gamma-PNAs [47]). Additionally, long homopyrimidine PNAs (>15 nucleotides) can form regular triplex structures (PNA-triplexes) at physiologically relevant salt concentrations [48], whereas stable PNA-triplex and triplex-invasion complexes have not been reported for homopurine PNAs.

Locked nucleic acid (LNA) is an RNA analogue having a 2′-oxygen and 4′-carbon-methylene linkage. The presence of this bridge promotes a conformational restriction in LNA containing oligonucleotides, favoring duplex formation [49]. LNA modification has also been introduced in TFOs to increase triplex stability. Like PNA, LNA is also able to invade dsDNA through Watson-Crick hydrogen bond formation to the DNA complementary sequences or through combined Watson-Crick and Hoogsteen hydrogen bonds forming a bisLNA construct, analogous to bisPNA [50].

In a previous study on the formation of triplex structures at FRDA (GAA)_n_ repeats, we showed that a low-molecular weight benzoquinoquinoxaline compound (BQQ), recognizes triplex structures formed at (GAA)_n_ repeats in plasmids. BQQ is a DNA intercalating compound that specifically binds and stabilizes triplex structures of both purine and pyrimidine motifs [51–54]. Furthermore, BQQ is cell permeable, and we have shown that the compound binds and stabilizes H-DNA structures formed in plasmids in growing *Escherichia coli* cells [55]. Additionally, we have converted BQQ to a triplex-specific cleaving agent (BQQ-OP) by conjugation to a 1,10-phenanthroline ligand [52]. In the presence of Cu^2+^ and a reducing agent BQQ-OP causes dsDNA cleavage specifically at the site of formation of a triplex, and we have previously demonstrated the ability of BQQ-OP to probe triplex formation of both H-DNA and TFO-directed triplex structures in plasmids *in vitro* [55,56].

Sequence-specific targeting of GAA repeats using synthetic single strand oligonucleotides (ONs) is an attractive approach to examine the molecular mechanisms of non-canonical DNA structure formation at FRDA repeat expansions. While modified ONs as mRNA targeting therapeutics have made major progress lately, also in relation to triplet-repeat diseases [57,58], successful DNA targeting of nucleotide repeats using ONs has not been reported. In this study we aimed to target the alternative DNA structures at FRDA (GAA)_n_ repeats using modified ONs. We chose to examine PNA and LNA binding to these repeats owing to their ability to both invade dsDNA and to form triplex structure and thereby interfere with the formation of H-DNA and other higher order structures in FRDA expanded repeats. BQQ-OP mediated triplex-specific cleavage of dsDNA and chemical modification of ssDNA regions using chloroacetaldehyde were used to characterize the DNA structures and PNA-DNA complexes formed in short, medium and long (GAA)_n_ repeats. To assess global structural changes in plasmids containing (GAA)_n_ repeats in the presence of LNA oligomers, we used Atomic Force Microscopy (AFM). Our results demonstrate that targeting GAA repeats with PNA or LNA oligomers can resolve higher order structures formed in FRDA expanded repeats.

## Materials and Methods

### Plasmids

(GAA)_n_-containing plasmids, pMP179, pMP178 and pMP141, hereafter referred to as pMP179(115 repeats), pMP178(75 repeats) and pMP141(9 repeats) to denote the number of repeats, were a kind gift from Prof. M. Pandolfo’s Laboratory. The plasmids are derived from pSPL3 and the inserts are all flanked by 352 and 256 bp of human genomic sequences 5′ and 3′ of the (GAA)_n_ repeat, respectively [8,13].

### PNA and LNA Oligomers

PNA oligomers were synthesized by solid phase Boc-chemistry, purified by RP-HPLC and characterized by MALDI-TOF mass spectrometry and HPLC [41]. LNA-PS oligomers were synthetized, purified by Reverse Phase - HPLC; quality control was accessed by MALDI-TOF mass spectrometry and bought from Eurogentec S.A.

### BQQ-OP Triplex-Specific DNA Cleavage of DNA-PNA Complexes at (GAA)_n_ Repeats

Plasmid pMP179(115 repeats) was linearized using ApaI, followed by DNA isolation using miniprep columns (Qiagen). 0.2 μg linearized pM179(115 repeats) (2.2 nM) was incubated at 37°C during 1 h in the presence of a 12-mer GAA-PNA (3320), a 15-mer CTT-PNA (3482) (10 μM) or a 20-mer single strand CTT-DNA (4 μM) (Table 1) in buffer (10 mM sodium cacodylate, 100 mM NaCl and 0, or 2 mM MgCl_2_, pH 7.5). BQQ-OP (1.5 μM) and CuSO_4_ (2 μM) were premixed at room temperature for 15 min and then added to the plasmid. The mixture was left for 25 min at room temperature and mercaptopropionic acid (MPA, 2 mM, final volume 20 μl) was added to initiate the cleavage reaction, which was allowed to proceed for 2 h at 37°C. Samples containing the crude reaction mixtures were then analyzed using 0.7% agarose gel electrophoresis (50 V, 1 h) and ethidium bromide staining. Gel-doc XR with Quantity One 4.5.2 software (Bio-Rad) was used for gel analysis and quantification of the gel bands. MassRuler (Fermentas) served as a molecular weight DNA ladder.

**Table 1:** PNA, DNA and LNA oligomers used in the study.

Name	Sequence	Length (nt)
**CTT-PNA (PNA3482)**	Ac-*TTCTTCTTCTTCTTC*-Eg1-Lys-NH_2_	15
**GAA-PNA (PNA3320)**	H-LysLys-*GAAGAAGAAGAA*-Lys-NH_2_	12
**CTT-DNA**	5′-cttcttcttcttcttcttct-3′	20
**GAA-DNA**	5′-agaagaagaagaagaagaag-3′	20
**GAA-LNA-PS 1**	5′-g*A*a*G*A*A*g*A*a-3′	9
**GAA-LNA-PS 2**	5′-g*A*a*g*A*a*g*A*a*g*A*a-3′	12
**GAA-LNA-PS 3**	5′-g*A*a*G*a*A*g*A*a*G*a*A-3′	12
**CTT-LNA-PS**	5′-c*T*t*C*t*T*c*T*t*C*t*T-3′	12
**SCR-LNA-PS**	5′-A*c*T*T*a*C*C*a*C*T*T*c-3′	12
**ER-LNA-PS**	5′-t*C*t*T*c*T*t*C*t*T*t*T-3′	12

*Italic* – *PNA*; lowercase – DNA; UPPERCASE – LNA and * – phosphorothioate backbone.

### BQQ-OP Mediated Cleavage of H-DNA at (GAA)_115_ Repeats in the Presence of PNA and LNA

Plasmid pMP179(115 repeats) (1 μg, 11 nM) was incubated in buffer (10 mM sodium cacodylate, pH 7.5 and 100 mM NaCl and 2 mM MgCl_2_) at 37°C during 2 h in the presence of 10 μM of either a 12-mer GAA-PNA (PNA3320), a 15-mer CTT-PNA (PNA3482), a 9-mer GAA-LNA-PS (GAA-LNA-PS 1), a 12-mer GAA-LNA-PS (GAA-LNA-PS 2 or 3), a 12-mer CTT-LNA-PS (CTT-LNA-PS), a 12-mer targeting the end of the repeat (ER-LNA-PS), a 20-mer single strand GAA-or a 20-mer CTT-DNA (Table 1). BQQ-OP (1 μM) and CuSO_4_ (1.5 μM) were premixed at room temperature (15 min) and then added to the plasmid solution. The mixture was left for 45 min at room temperature and mercaptopropionic acid (MPA, 2 mM, final volume 20 μl) was added to initiate the cleavage reaction. The reaction was allowed to proceed for 3 h at 37°C, followed by isolation of DNA using miniprep columns (Qiagen). As a control, plasmid pMP179(115 repeats) was cleaved in the absence of ONs using BQQ-OP under similar experimental conditions. The isolated DNA was digested using ApaI (1 U, Promega) during 3 h at 37°C and then analyzed using 0.7% agarose gel electrophoresis (50 V, 1 h) and ethidium bromide or SYBR-safe (Life Technologies) staining. Gel-doc XR with Quantity One 4.5.2 software (Bio-Rad) was used for gel analysis and quantification of the gel bands.

### Analysis of DNA and DNA-PNA structure formation at short and long (GAA)_n_ repeats using BQQ-OP cleavage and CAA modification

In all probing reactions, the plasmid (pMP141(9 repeats): 1 μg, 11.5 nM or pMP178(75 repeats): 11.2 nM) was first incubated in buffer (10 mM sodium cacodylate, 100 mM NaCl, 2 mM MgCl_2_, pH 7.5) at 37°C, 2 h in the absence or presence of either 12-mer GAA-PNA or 15-mer CTT-PNA (10 μM) (Table 1). PNA binding was followed by BQQ-OP mediated DNA cleavage or DNA chemical modification using chloroacetaldehyde (CAA).

*BQQ-OP cleavage*: BQQ-OP (1 μM) and CuSO_4_ (1.5 μM) were premixed (15 min) at room temperature and added to the PNA-plasmid solution. The mixture was left for 45 min at room temperature and mercaptopropionic acid (MPA, 2 mM, final volume 20 μl) was added to initiate the cleavage reaction. The reaction was allowed to proceed for 3 h at 37°C, followed by isolation of DNA using miniprep columns (Qiagen). The isolated DNA was digested using ApaI (Fermentas Fastdigest) and the enzyme was then inactivated, 5 min at 65°C.

*CAA chemical modification*: CAA (2%) was added to the plasmid solution (final volume 20 μl) and the reaction was allowed to proceed during 30 min at 37°C, followed by isolation of the DNA using miniprep columns (Qiagen). Samples incubated under similar conditions in the absence of CAA were used as controls. The isolated DNA was digested as described in the previous section.

*Primer extension*: The primer pair pMP1764F (5′-CTCTGGAGTAGCTGGGATTACAG-3′) and pMP1333R (5′-CCAACATGGTGAAACCCAGTATCTAC-3′) were 5′-radioactively labeled using [γ-^32^P]ATP and T4 polynucleotide kinase (Fermentas) according to the manufacturer’s protocol and subsequently purified using a QIAquick Nucleotide Removal Kit (QIAGEN). Plasmids treated by BQQ-OP or CAA were used as templates. A primer extension (PE) mix (2 mM MgCl_2_, 1 U taq polymerase (Fermentas), 5 nM primer, 2 mM of each dNTP) was added to approximately 100 ng template and a PE reaction was carried out according to the following condition; 10 min at 94°C, 30 cycles of 1 min at 94°C, 2 min at 54°C or 49°C (primer pMP1333R and pMP1764F, respectively), 3 min at 72°C, and 10 min at 72°C. As controls for the PE reactions, plasmids were incubated under similar conditions in the absence of BQQ-OP or CAA. Sequencing ladders were prepared by using plasmids (100 ng) that had been cleaved by using PstI and SacI as templates for the PE reactions in the presence of dideoxynucleotides as described in the literature [59]. All samples were analyzed using denaturing polyacrylamide gel electrophoresis (6%, 7 M urea, 0.5 mm) in buffer (1X TBE) at room temperature and 1200 V, 32 mA, 2.5 h. Fuji FLA3000 phosphorimager was used for scanning, analysis and gel bands quantification.

### AFM Analysis of pMP179(115 Repeats) in the Presence of LNA

Plasmid pMP179(115 repeats) (500 ng) was incubated in intranuclear buffer (final concentration: 50 mM Tris-acetate, 120 mM KCl, 5 mM NaCl, 0.5 mM Mg-acetate and pH 7.4) at 37°C, overnight and in the absence or presence of 10 μM of either GAA-LNA-PS 3, CTT-LNA-PS-or a 12-mer scrambled phosphorothioate LNA (SCR-LNA-PS) (Table 1), after which the samples were immediately frozen until further use. Sample preparations prior the AFM measurements were identical for every treatment, where, after de-frost, 5 μL of each plasmid preparation (2.5 ng/μL) was mixed with 25 μL of 20 mM NiCl_2_.5H_2_O solution (Sigma-Aldrich) and 25 μL of 10 mM 4-(2-hydroxyethyl)-1-piperazineethanesulfonic acid buffer, pH 7.2 (HEPES, Sigma-Aldrich), in a similar way as described by Pyne et *al*. [60]. The mixture was deposited on a freshly cleaved mica surface (Sigma-Aldrich) and incubated during 30 minutes at room temperature. The sample was then diluted with 50 μL of 10 mM HEPES buffer, pH 7.2, which reduces the NiCl_2_ concentration to 5 mM. Subsequently, the mica surfaces were analyzed in the liquid solution. PeakForce^®^ tapping mode (PTM) measurements were performed on an atomic force microscope (Dimension Fast Scan, Nanoscope V, Bruker^®^) located in the AlbaNova Nanofabrication Facility (Stockholm, Sweden). In these measurements the scanner vibrates at a low frequency (1–3 kHz), resulting in a tip-sample interaction with every oscillation. The maximum force applied was kept constant with a feedback loop, which adjusts the overall extension of the piezo during scanning. This allows direct control of the force exerted, which is not the case for the traditional tapping mode (TM) [61,62]. In this work, the AFM images were obtained using silicon nitride cantilevers with silicon tips (ScanAsyst-Fluid^+^, Bruker^®^) with a tip nominal radius of 2 nm and spring constants ranging between 0.4 and 0.7 Nm^-1^. The images presented in this work were obtained with scan rates between 1 and 1.5 Hz, maximum force of 500 pN (lowest force possible to achieve due to optical interference), scanner oscillation amplitudes between 60–40 nm, scanner resonance frequency of 2 kHz, image resolution of 512 x 512 pixels and scan sizes around 5 x 5 and 1.3 x 1.3 μm^2^. In all the images, the vertical limit was reduced to 1 μm in order to enhance the resolution. Plasmid areas from all scans obtained were calculated by Image J software and GraphPad Prism 6 software was used for statistical analysis.

### Statistical Analysis

Data is presented as means ± S.D. or mean ± S.E.M., when indicated. Values were tested for normality by the D’Agostino-Pearson normality test (omnibus K2). Statistical significance was determined by one-way ANOVA two-sided, followed by comparison of each treatment with the group control by Fisher’s Least Significance Difference (LSD) test (Graph Pad Prism 6 Software, Graph Pad Software, Inc.). In all cases *P*<0.05 was considered significant.

## Results and Discussion

### Formation of PNA-Directed Triplex at (GAA)_n_ Repeats

We previously demonstrated that homopyrimidine natural DNA TFOs bind dsDNA at GAA repeat sequences forming a pyrimidine motif triplex. Binding occurs with moderate efficiency at physiological pH. Conversely, purine motif triplex formation was not detected at this site when using the corresponding homopurine TFO [56]. Here, we aimed to test whether PNA would behave differently when targeted to FRDA expanded GAA repeats. PNA oligomers consisting of CTT or GAA repeat sequences were used (Table 1, CTT-PNA and GAA-PNA, respectively). Triplex formation by PNA oligomers was examined using a triplex-specific dsDNA cleavage reaction mediated by BQQ-OP [52]. However, BQQ-OP cleavage of triplex DNA does not discriminate between H-DNA formed inherently by the FRDA (GAA)_n_ repeats and a TFO-directed structure. Because H-DNA only readily forms in supercoiled plasmids, linearization of the GAA carrying plasmid by a unique site restriction enzyme is required before carrying out the BQQ-OP cleavage assay to ensure that only the intermolecular PNA-triplex (and not H-DNA) would be formed and detected.

**Fig 2:**
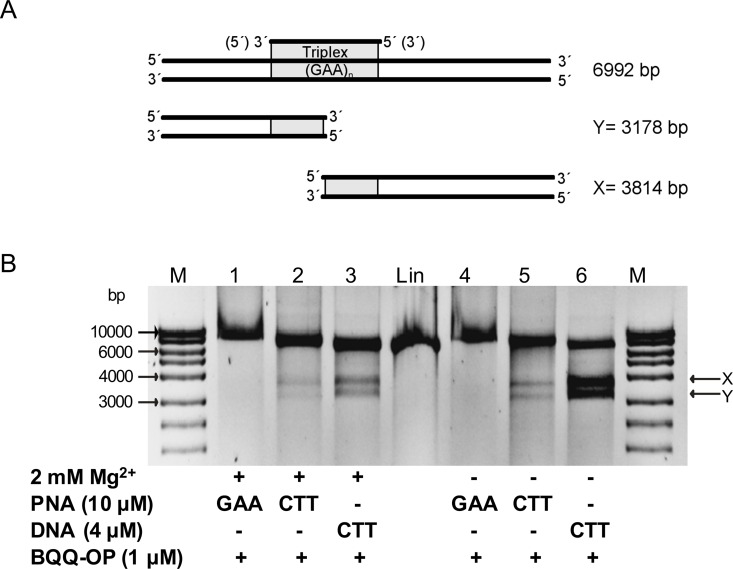
Triplex-specific DNA cleavage of PNA triplex at (GAA)_115_ repeats in linearized pMP179. **A)** Schematic presentation of TFO-directed triplex formation and the two fragments generated after BQQ-OP cleavage indicated as X (3814 bp) and Y (3178 bp). **B)** Representative gel of linearized pMP179(115 repeats) (Apa1) incubated with GAA-PNA (10 μM), CTT-PNA (10 μM) or CTT-DNA (4 μM) (Table 1) in buffer (10 mM sodium cacodylate, 100 mM NaCl and 0 or 2 mM MgCl_2_, pH 7.5), as indicated. BQQ-OP mediated cleavage of pMP179(115 repeats) was carried out in the presence of Cu^2+^ and MPA. Reference linearized pMP179(115 repeats) (Lin) and a molecular weight DNA ladder (M) are also shown.

We used plasmid pMP179(115 repeats), which contains 115 GAA repeats and flanking sequences derived from the first intron of the *FXN* gene. After linearization with ApaI, the plasmid was incubated with CTT-PNA or GAA-PNA and cleaved by BQQ-OP. As control a triplex was also formed in the presence of a single strand CTT-DNA (Table 1). If dsDNA cleavage by BQQ-OP would occur specifically at the site of triplex formation, the reaction should result in the formation of two DNA fragments. The size of these fragments is approximately 3814 and 3178 bp, assuming an average triplex cleavage to occur in the middle of the triplex forming (GAA)_115_ repeat (Fig 2A). Binding of CTT-PNA or CTT-DNA followed by BQQ-OP dsDNA cleavage resulted in the formation of two DNA fragments having the expected sizes (Fig 2B, lanes 2 and 5 or 3 and 6, respectively). A stronger intensity of the bands corresponding to the BQQ-OP cleavage was seen when CTT-DNA was used compared to conditions obtained by CTT-PNA binding, indicating either stronger binding of the DNA (although PNA-DNA_2_ triplexes of identical length are much more stable than DNA triplexes [63]) or more efficient BQQ-OP cleavage of the DNA triplex versus the PNA-DNA_2_ triplex. The same trend was observed both in the presence as well as the absence of Mg^2+^ ions. However, it is well known that an additional PNA-DNA complex, a PNA-triplex invasion, can be formed when homopyrimidine PNA are targeted to polypyrimidine-polypurine dsDNA, and this is more stable than a PNA-triplex [64]. In the case of PNA-triplex invasion, only the GAA DNA strand is engaged in the triplex while the other strand is displaced. Therefore, BQQ-OP would only lead to a single strand cleavage of the plasmid. Hence, the resulting DNA fragment would be of the same size as the linearized plasmid (Lin), which can be seen in Fig 2B (lanes 2 and 5).

Next, we examined the ability of GAA-PNA to form a purine motif intermolecular triplex using the BQQ-OP dsDNA cleavage assay. We detected only a single DNA fragment exhibiting slightly slower gel mobility than the linearized plasmid (Fig 2B, lanes 1 and 4), which could indicate stable PNA binding to the dsDNA plasmid, maybe due to formation of a duplex invasion complex. Nevertheless, triplex formation in the presence of GAA-PNA was not detected using the BQQ-OP assay. Interestingly, we did not observe a purine motif TFO-DNA triplex nor a purine motif H-DNA in earlier studies [56]. Based on these observations and that the triplex formed by CTT-PNA binding resulted in a clear BQQ-OP cleavage, we conclude that a purine motif triplex is not formed in the presence of GAA-PNA.

### PNA Binding to H-DNA Forming FRDA (GAA)_115_ Repeats

**Fig 3:**
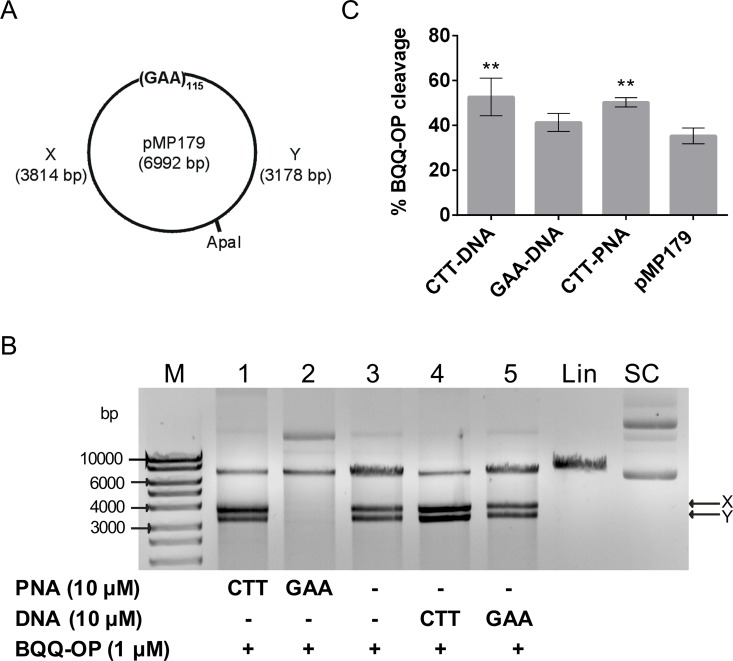
BQQ-OP mediated DNA cleavage of H-DNA forming (GAA)_115_ repeats in the presence of PNA. **A)** Schematic presentation of pMP179 and the H-DNA forming site. The two DNA fragments generated by triplex-specific cleavage followed by enzymatic digestion are indicated as X (3814 bp) and Y (3178 bp). **B)** Representative gel for pMP179(115 repeats) incubated with 10 μM PNA (CTT-PNA or GAA-PNA, lane 1 or 2, respectively), in the absence of ONs (lane 3) or with 10 μM ssDNA (CTT-DNA or GAA-DNA, lane 4 or 5, respectively) in buffer (10 mM sodium cacodylate, 100 mM NaCl, 2 mM MgCl_2_, pH 7.5). Triplex-specific cleavage was carried out in the presence of Cu^2+^ and MPA, followed by digestion with ApaI. Reference supercoiled (SC) and linearized (Lin) pMP179(115 repeats) and a molecular weight DNA ladder (M) are also shown. **C)** Graph represents the percentage of BQQ-OP mediated cleavage obtained for pMP179(115 repeats) in the absence (indicate in the graph as pMP179) or in the presence of PNA- or DNA oligomers. Values indicate the ratio between the intensity of DNA double strand cleavage to the total band intensity from the respective lane and are expressed as mean±S.D. (n=3). No cleavage was obtained in the presence of GAA-PNA and therefore not included in the graph. ** *P*≤0.01 in relation to plasmid in the absence of oligonucleotide (pMP179).

H-DNA is found in two different motifs (parallel pyrimidine or antiparallel purine triplex) and both have been proposed to form at FRDA (GAA)_n_ repeats (Fig 1). We recently showed that expanded (GAA)_115_ repeats form a pyrimidine motif triplex in supercoiled plasmids by using the BQQ-OP assay [56]. Also, binding of a single strand CTT oligonucleotide (identical in sequence and length to the currently used CTT-DNA, Table 1) to the H-DNA forming plasmid enhanced triplex-formation significantly, whereas an analogous GAA oligonucleotide (corresponding to GAA-DNA, Table 1) had no such effect. These findings prompted us to examine whether binding of modified ON such as CTT-PNA or GAA-PNA (Table 1) to the (GAA)_115_ repeats would have a comparable effect on H-DNA formation at this site. When BQQ-OP mediated dsDNA cleavage occurs within the (GAA)_115_ triplex-forming repeat, the subsequent unique site enzymatic digestion is expected to generate two linear DNA fragments of approximately 3814 and 3178 bp (as shown in Fig 3A). In the absence of DNA or PNA oligomers, dsDNA cleavage by BQQ-OP, corresponding here only to inherent H-DNA formation, was estimated, by quantification of the gel bands (Fig 3B, lane 3), to be on average 35.3±3.5% (Fig 3C). On the other hand, binding of CTT-PNA to the (GAA)_115_ repeat leads to a statistically significant increase of the amount of triplex, as measured by the extent of BQQ-OP mediated DNA cleavage (50.3±2.1%, *P*=0.0068) (Fig 3B, lane 1 and Fig 3C). As previously reported, the presence of CTT-DNA also leads to a significant increase in the level of BQQ-OP cleavage to 52.7±8.4%, *P*=0.0031 (Fig 3B, lane 4 and Fig 3C). Taken together, our findings suggest that sequence-specific binding of CTT-PNA to FRDA expanded repeats enhances triplex formation under these conditions.

We envision three models to account for these results. Either the H-DNA structure is stabilized by PNA binding to the single stranded GAA loop formed as part of the H-DNA structure or the H-DNA is replaced by a PNA-DNA_2_ triplex, or a PNA_2_-DNA triplex invasion structure over the entire GAA repeat. However, under similar binding and cleavage conditions using the linearized dsDNA we detected only TFO-triplex formation at a low level in the presence of CTT-PNA (Fig 2B, lane 2), which suggests rather weak TFO-binding. Thus, it is most likely that the enhancement of triplex-directed DNA cleavage by BQQ-OP, as shown in Fig 3B (lane 1 and 4), reflects mainly a stabilization of the H-DNA structure through binding of CTT-DNA or CTT-PNA (via a PNA_2_-DNA triplex structure) to the single stranded GAA repeat region of the H-DNA.

Interestingly, when we targeted FRDA repeats in pMP179(115 repeats) using a GAA-PNA oligomer (Table 1) there was no detectable triplex-containing structure, including H-DNA, (Fig 3B, lane 2). Only one major DNA fragment was observed upon BQQ-OP treatment, corresponding in size and gel mobility to the linearized plasmid. These results indicate a completely different behavior of the GAA-PNA when interacting with the *FXN* triplet repeats as compared to GAA-DNA (Fig 3B, lane 5). Apparently, GAA-DNA does not bind to the repeat region and consequently H-DNA is still formed to a comparable extent as in the control sample (41.3±4.0%, Fig 3B, lane 3 and Fig 3C). It is worth noting that a slower-mobility band is detected in the sample containing the (GAA)_115_ repeat plasmid and GAA-PNA (Fig 3B, lane 2). The presence and intensity of this band indicates a stable interaction, which we believe can be attributed to a sequence-specific GAA-PNA binding to (GAA)_115_ repeats in the plasmid. However, this assay cannot unravel the nature of this interaction, which called for further detailed analysis as described in the following section. Nevertheless, our findings show that binding of a GAA-PNA oligomer to FRDA repeats has the unique ability to completely abolish all triplex structures, which are detected by BQQ-OP, including H-DNA, under these conditions.

### Structural Analysis of CTT-PNA and GAA-PNA Binding to (GAA)_n_ Repeats

To better understand the binding modes of CTT-PNA and GAA-PNA oligomers to the FRDA expanded (GAA)_n_ repeats and the effect of PNA binding on H-DNA, or other higher order structure formation at these expansions, we examined PNA binding into two different supercoiled plasmids carrying short or medium (GAA)_n_ repeats (n=9 or 75, respectively) and including FRDA flanking sequences. We employed structural probing analysis using either chemical modification of ssDNA regions by chloroacetaldehyde (CAA) or BQQ-OP mediated cleavage of DNA triplex structures. Chemical modifications of DNA and triplex-specific cleavage by BQQ-OP were analyzed using primer extension (PE) reactions where each of the (GAA)_n_ or (CTT)_n_ containing strands, which are referred to as the R-strand and the Y-strand, respectively, were used as template. Chloroacetaldehyde reacts with single strand adenosines and cytosines, which prevents PE by DNA polymerase. BQQ-OP cleavage, on the other hand, is used to map regions of triplex formation. Both probing reactions were carried out in the absence or presence of PNA (CTT-PNA or GAA-PNA). For all DNA reactions, we included a control sample, where the plasmid was incubated in the absence or presence of PNA but no DNA chemical modification or BQQ-OP cleavage reaction was carried out (control C1, C2, and C3). When analyzing DNA cleavage or chemical modification, the intensity of each band was compared to the corresponding band in the control sample. This is necessary to identify background signals, which are not related to any specific DNA modification or cleavage but result from DNA polymerase pausing/arrest of PE at PNA binding sites, or by formation of stable structures, in the DNA template.

### H-DNA Formation at Short (GAA)_9_ Repeats

**Fig 4:**
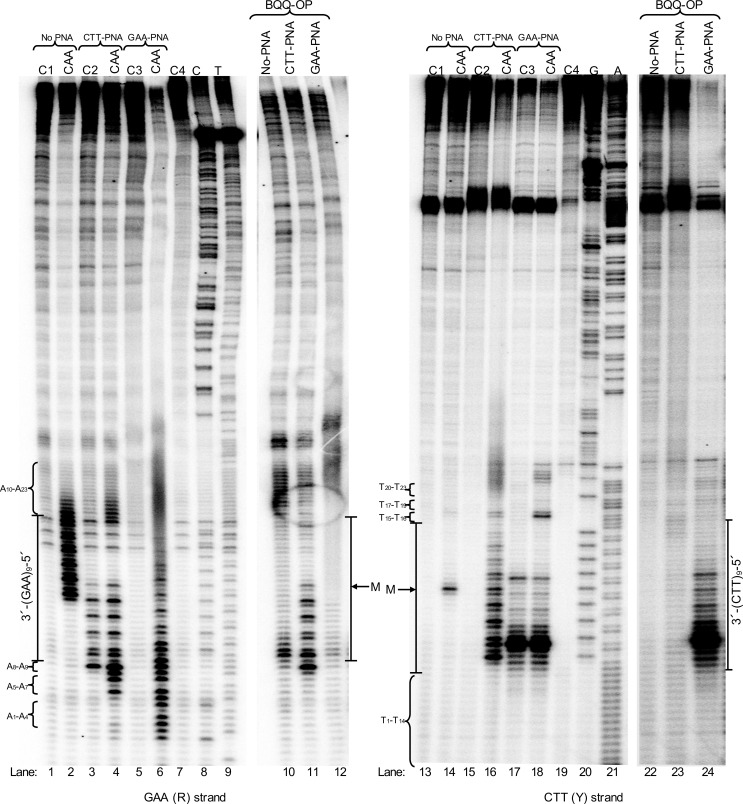
Structural- and chemical probing of DNA and DNA-PNA complex formation at (GAA)_9_ repeats. Non-denaturing PAGE of DNA fragments mapped by BQQ-OP cleavage and chloroacetaldehyde (CAA) modification followed by primer extension (PE). Plasmid pMP141(9 repeats), was incubated in the absence (No PNA) or presence of 10 μM of GAA-PNA or CTT-PNA in buffer (10 mM sodium cacodylate, 100 mM NaCl, 2 mM MgCl_2_, pH 7.5). The plasmid was then either chemically modified using 2% CAA or cleaved using 1 μM BQQ-OP. Untreated plasmid was used in a set of four different controls (C) of the PE reactions: C1= plasmid incubated in the absence of PNA. C2= plasmid incubated in the presence of CTT-PNA. C3= plasmid incubated in the presence of GAA-PNA. C4= plasmid not incubated. All samples were linearized using ApaI and then used as templates for the PE reaction. Sequence ladders using dideoxynucleotides (C=ddCTP, T=ddTTP, G=ddGTP and A=ddATP), linearized pMP141(9 repeats) and a PE reaction control (C4) are also shown. The left gel panel shows the DNA fragments obtained in a PE reaction using the GAA containing (R)-strand as template, and the right gel panel shows the DNA fragments obtained when using the CTT containing (Y)-strand as template. The A_1_ to A_23_ nucleotides flanking the repeats of the R-strand and T_1_ to T_23_ flanking in the Y-strand and the mid-point of the repeat sequence (M) are indicated.

**Fig 5:**
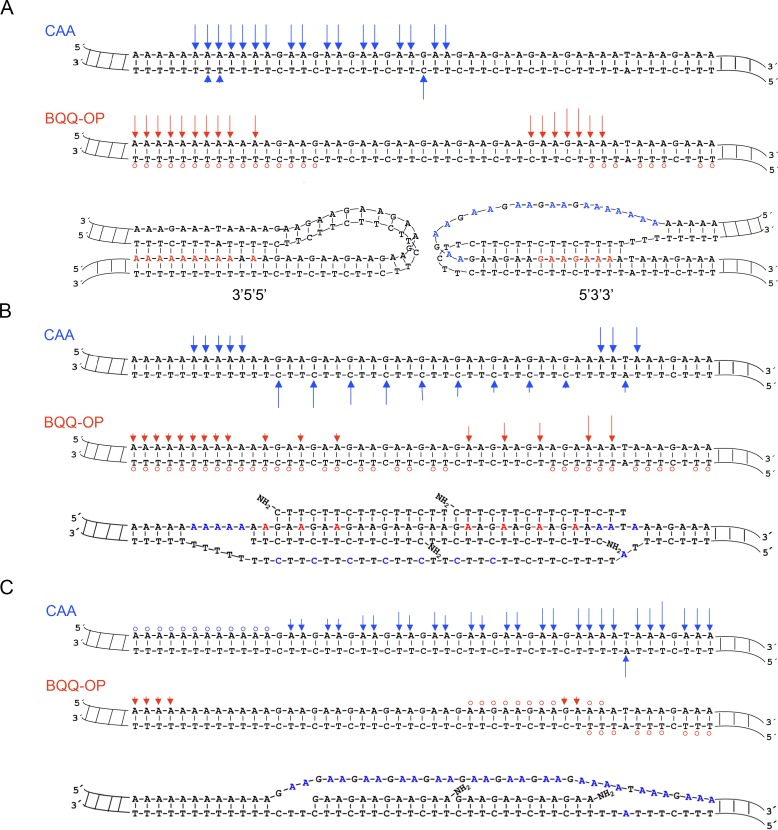
Models showing the most predominant DNA and DNA-PNA structures formed at pMP141(9 repeats) in the absence or the presence of CTT-PNA or GAA-PNA respectively. **A**) The H-DNA structure (lower panel, right side structure) corresponds to the predominant structure formed in the absence of PNA. The H-DNA structure (lower panel, left side) corresponds to the putative 3′5′5′-isomer of the H-DNA. **B)** The triplex-invasion structure (lower panel) corresponds to the predominant structure formed in the presence of CTT-PNA. **C)** The duplex-invasion structure (lower panel) corresponds to the predominant structure formed under these conditions. In **A**, **B** and **C)** CAA modifications and BQQ-OP cleavage sites of pMP141(9 repeats) in the absence or presence of PNA are indicated in the DNA duplex as blue (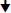) and red (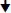), respectively and circles are used to mark a very low level of modification or cleavage. The height of the arrow represents the relative within-lane intensities of gel bands corresponding to CAA modifications and BQQ-OP cleavage. The nucleotides marked in blue or red indicate the sites of chloroacetaldehyde DNA modification or BQQ-OP mediated DNA cleavage, respectively. All indicated modifications, of samples treated with BQQ-OP or CAA, are compared to the controls.

Chloroacetaldehyde probing clearly demonstrates the presence of a single strand region starting in the middle of the (GAA)_9_ repeat and covering the 5′-half of the repeat region (Fig 4, R-strand, lane 2). Analysis of the corresponding Y-strand, only revealed a very short (1–3 nt) single strand region (Fig 4, Y-strand, lane 14). This region is localized in the middle of the mirror repeat (GAA)_9_ sequence, which is consistent with formation of a short loop in a 5′3′3′-pyrimidine motif H-DNA. Fig 5A (upper panel) shows the sites of chloroacetaldehyde modifications (indicated by blue arrows) and the corresponding H-DNA (lower panel, right side).

BQQ-OP probing of triplex regions of the R-strand showed that cleavage occurred mainly at the 3′-end of the repeat and also extended to the shorter A-rich (A_5_ – A_9_) flanking region (Fig 4, lane 10). Moreover, cleavage is also detected at the 5′-end flanking nucleotides corresponding to the longer A-rich region (A_10_ – A_23_). The results suggest engagement of the R-strand of the repeat in more than one triplex structure or H-DNA isomer. Analysis of BQQ-OP cleavage of the Y-strand shows no significant cleavage (Fig 4, lane 22) when compared to cleavage of the R-strand. We previously reported that intercalation of BQQ-OP in pyrimidine motif triplex DNA and the corresponding *in situ* radical reaction results in a lower rate of DNA cleavage of the pyrimidine-rich strand (corresponding here to the Y-strand) and a more pronounced cleavage of the purine-rich strand (corresponding here to the R-strand) [52]. BQQ-OP mediated cleavage is illustrated in Fig 5A (middle panel) and the site and intensity of cleavage is indicated by red arrows (higher level of cleavage) or circles (lower level). The combined chloroacetaldehyde and BQQ-OP probing results are in accordance with previous reports [9,56] and the proposed 5′3′3′ H-DNA structure is based on these results (lower panel, right side).

However, triplex-directed dsDNA cleavage using BQQ-OP indicates the formation of two different H-DNA pyrimidine motif triplexes, but only one H-DNA isomer (Fig 5A, lower panel, right side), corresponding to a 5′3′3′ H-DNA is also supported by the chloroacetaldehyde probing results (Fig 4, lane 2), where a clear single strand formation is demonstrated. The different time kinetics of the chloroacetaldehyde reaction (30 min) and the BQQ-OP triplex-specific cleavage (3 h) could (partly) provide an explanation. It has been shown that the kinetics of H-DNA formation is different for each of the two isomers (5′3′3′ and 3′5′5′). Roberts and Crothers reported, in a study using palindromic pyrimidine strands, which can fold into a triplex structure upon binding of a complementary purine strand, that the 5′3′3′-isomer of the intramolecular triplex folds 10–50 times faster than the 3′5′5′ isomer even though both isomers are equally stable as final complexes [65]. Consequently, it is possible that chloroacetaldehyde probing reveals only formation of the kinetically favored 5′3′3′ H-DNA isomer, whereas the much slower BQQ-OP cleavage detects both isomers. This suggestion is supported by the fact that the intercalating moiety in BQQ, BQQ-OP and other BQQ-conjugates has a higher affinity to T.AxT stretches, as compared to C.GxC triads [52–54], which is the case here including the 5′-end flanking sequence of the R-strand. Therefore, the results of BQQ-OP cleavage could correspond to the formation of an additional H-DNA isomer (Fig 5A; lower panel, left side).

### Probing PNA Binding at Short (GAA)_9_ Repeats

PNA binding to the (GAA)_9_ repeats in pMP141(9 repeats) was analogously studied using chloroacetaldehyde modification and BQQ-OP cleavage.

#### Binding of GAA-PNA

Chloroacetaldehyde reaction of the GAA-PNA treated pMP141(9 repeats) showed that a single strand region is formed throughout the entire repeat and flanking sequences of the R-strand (Fig 4, lane 6) as compared to control samples that were not subjected to chemical modification (Fig 4, lanes 1 and 5). In contrast, only a few single strand nucleotides were identified at the 5′-end flanking sequence of the repeat of the Y-strand (Fig 4, lane 18, T_15_-T_16_) as compared to control lanes (Fig 4, lane 13 and 17). Only very weak bands on both the R- and Y-strands were obtained from the BQQ-OP cleavage of the GAA-PNA treated plasmid, pMP141(9 repeats) (Fig 4, lane 12 and 24, respectively), as compared to control lanes (Fig 4, lane 5 and 17, respectively). This together with a clear formation of single strand region throughout the R-strand is therefore ascribed to GAA-PNA duplex invasion of the repeat region, as illustrated in the proposed model structure in Fig 5C (lower panel).

#### Binding of CTT-PNA

Chloroacetaldehyde treatment of the R-strand in the presence of CTT-PNA indicated two short single strand sites at the 3′- and 5′-end flanking nucleotides within the A-rich regions (A_5_ – A_9_ and A_10_ – A_23_) (Fig 4, lane 4). As mentioned previously, a control sample of the PNA binding plasmid is included to indicate bands that are present due to polymerase pausing at the binding site (Fig 4, lane 3). Furthermore, analysis of the Y-strand, following incubation with CTT-PNA revealed a single strand region that covers the whole (CTT)_9_ repeat sequence (Fig 4, lane 16). The gel also shows that the band intensity in lane 16 gradually increases towards the 3′-end of the repeat. Again, a control sample consisting of a CTT-PNA treated plasmid was included (Fig 4, lane 15). The results are compatible with the formation of a triplex invasion complex (Fig 5B, lower panel) of two CTT-PNA oligomers binding to the R-strand at the (GAA)_9_ repeats in pMP141(9 repeats). The prevalence of this structure is also supported by BQQ-OP mediated triplex-specific cleavage (Fig 4, lane 11), which shows only very little additional cleavage as compared to the control sample (Fig 4, lanes 3), possibly indicating remaining H-DNA. Taken together, our results indicate that CTT-PNA binding to the repeat region in pMP141(9 repeats) favors formation of a triplex-invasion.

### Formation of Stable H-DNA Structure(s) at Medium (GAA)_75_ Repeats

**Fig 6:**
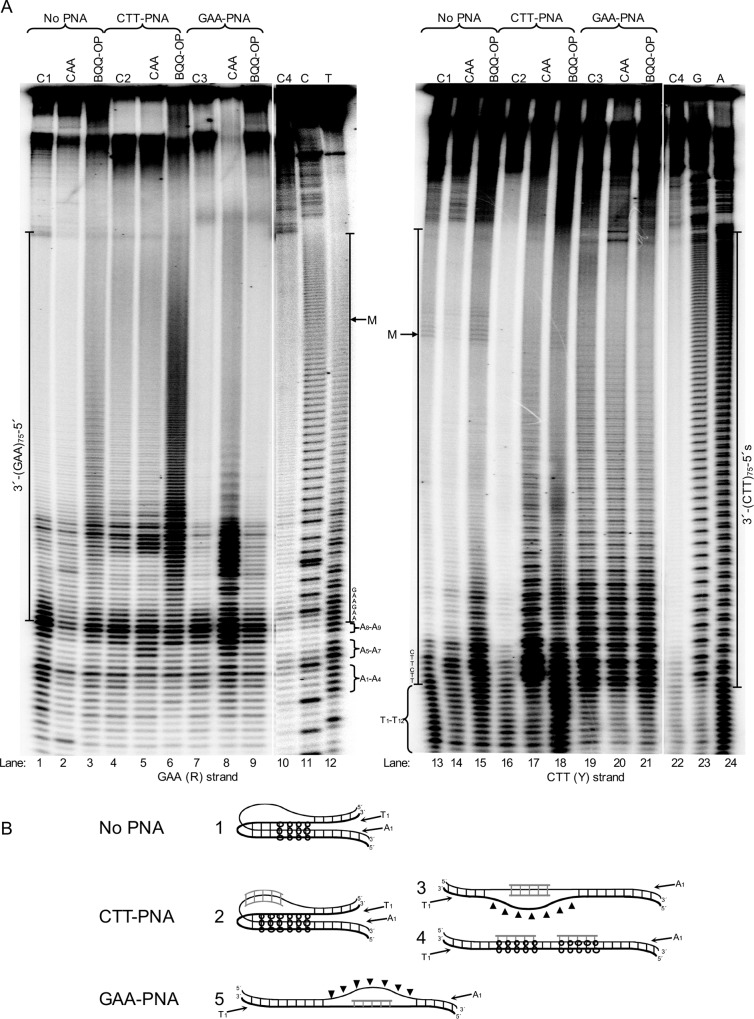
Structural- and chemical probing of DNA and DNA-PNA complex formation in pMP178(75 repeats). **A)** Non-denaturing PAGE of DNA fragments mapped by BQQ-OP cleavage and chloroacetaldehyde (CAA) modification followed by primer extension (PE). Plasmid pMP178(75 repeats), was incubated in the absence (No PNA) or the presence of 10 μM of PNA (GAA-PNA or CTT-PNA) in buffer (10 mM sodium cacodylate, 100 mM NaCl, 2 mM MgCl_2_, pH 7.5). The plasmid was then chemically modified using 2% CAA or cleaved using 1 μM BQQ-OP. Non-treated plasmid was used in a set of four different controls (C) of the PE reactions C1= plasmid incubated in the absence of PNA. C2= plasmid incubated in the presence of GAA-PNA. C3= plasmid incubated in the presence of CTT-PNA. C4= plasmid not incubated. All samples were linearized by ApaI then subjected as templates for the PE reaction. Sequence ladders using dideoxynucleotides (C=ddCTP, T=ddTTP, G=ddGTP and A=ddATP), linearized pMP178(75 repeats) and the PE reaction control (C4) are shown as references. The DNA fragments detected in a PE reaction using the GAA (R)-strand as template, and the right gel panel shows the DNA fragments detected in a PE reaction using the CTT (Y)-strand as template. The A_1_ to A_9_ nucleotides flanking the repeats of the R-strand and T_1_ to T_12_ flanking in the Y-strand and the middle point of the repeat sequence are indicated (M). **B)** Models showing the most predominant DNA and DNA-PNA structures formed at pMP178(75 repeats) in the absence or the presence of CTT-PNA or GAA-PNA respectively. They correspond to: 1. 5′3′3′-pyrimidine motif H-DNA, 2. CTT-PNA stabilized 5′3′3′-pyrimidine motif H-DNA, 3. Triplex-invasion, 4. Intermolecular triplex and 5. Duplex-invasion structure. Thin line= purine strand, thick line= pyrimidine strand, grey line= PNA. Regions modified by CAA (π) or cleaved by BQQ-OP (o) are indicated.

Pathological GAA repeat expansions in FRDA may vary in length and structural probing of long DNA sequences has proven challenging. Therefore, we chose to examine H-DNA and higher order structure formation in a medium GAA repeats using an FRDA derived DNA sequence that consists of 75 repeats and genomic flanking regions and we attempted to map formation of single- and triple strand regions using chloroacetaldehyde and BQQ-OP, respectively. Analysis of the R- and Y-strands that were subjected to BQQ-OP probing clearly indicated the presence of triplex structures as judged from the gel bands corresponding to DNA cleavage within the repeat region (Fig 6A, lanes 3 and 15). However, the band intensity in lane 3 shows that BQQ-OP mediated DNA cleavage is more pronounced at the 3′-end of the R-strand indicating formation of a 5′3′3′- pyrimidine H-DNA (Fig 6B, structure 1). Reaction of the Y-strand also indicates some preference at the 3′-end. In contrast to the clear single strand probing of the GAA strand within the short repeat (Fig 4), chloroacetaldehyde modification of the longer repeat failed to reveal bands that could indicate the presence of non base-paired nucleotides in either of the R- or Y-strand. These findings may reflect the presence of more complex higher order DNA structures at the medium GAA repeats indicating a difference between the pathological and normal repeat lengths.

### The Effect of GAA-PNA Binding on H-DNA Formation at (GAA) _75_ Repeats

Binding of GAA-PNA to the (GAA)_75_ repeats in supercoiled plasmid was examined using chloroacetaldehyde and BQQ-OP reactions. Binding of GAA-PNA resulted in a clear and strong chloroacetaldehyde reaction with the R-strand providing evidence for a single strand formation in particular at the 3′-end of the GAA repeat (Fig 6A, lane 8), whereas reaction above background could not be detected within the Y-strand (Fig 6A, lane 20) under similar conditions. Most interestingly, no BQQ-OP mediated triplex-specific cleavage of the R- or Y-strand was seen when the plasmid was incubated with GAA-PNA (Fig 6A, lanes 9 and 21 compared to the control lanes 7 and 19, respectively).

The findings from chemical probing of the DNA structures formed using GAA-PNA targeting of medium GAA repeats in pMP178(75 repeats) (Fig 6) are consistent with those obtained when probing the short GAA repeat, pMP141(9 repeats) (Fig 4 and Fig 5). Together they demonstrate that binding of GAA-PNA to GAA repeats results in formation of a duplex invasion complex (Fig 6B, structure 5), which can explain the altered mobility of the linear fragment seen in the presence of GAA-PNA in Fig 2 (lane 1 and 4). This in turn could prevent the expanded repeats from forming an H-DNA or any other higher order structure, including triplex. The results here are in complete agreement with the lack of BQQ-OP cleavage in the GAA-PNA treated pMP179(115 repeats) as detected using agarose gel electrophoresis (Fig 3B, lane 2).

To the best of our knowledge, this is the first study that demonstrates formation of a duplex invasion complex, which forms through binding of GAA-PNA to expanded FRDA repeats, thereby dissolving a triplex containing higher order structure.

### The Effect of CTT-PNA Binding on H-DNA Formation at (GAA) _75_ Repeats

Based on the results of H-DNA probing and the outcome of PNA binding of pMP179(115 repeats) (Fig 3), we concluded that the presence of CTT-PNA enhances triplex formation within the repeat sequence. Therefore, we examined how this PNA oligomer affects the dsDNA medium repeat region by using the single strand and triplex region probing assays.

Targeting the (GAA)_75_ repeats in pMP178(75 repeats) with CTT-PNA resulted in an enhanced triplex-directed cleavage by BQQ-OP of the R-strand (Fig 6A, lane 6) as compared to the control (lane 3). This result quantitatively agrees with triplex-specific cleavage at (GAA)_115_ repeats, analyzed using agarose gel electrophoresis (Fig 3B, lane 1). In addition, an analogous enhancement was seen for the Y-strand (Fig 6A, lane 18 compared to lane 15).

Chemical modification using chloroacetaldehyde of the pMP178(75 repeats) in the presence of CTT-PNA did not detect any major single-strand regions in the R-strand (Fig 6A, lane 5 as compared to control lane 4), as also observed in the absence of PNA (Fig 6A, lane 2 as compared to control lane 1). However, chloroacetaldehyde probing of the Y-strand in pMP178(75 repeats) in the presence of CTT-PNA strongly indicates the formation a single strand region that extends to nearly half the length of the mirror repeat sequence (towards the 3′-end) (Fig 6A, lane 17). Since homopyrimidine PNA has the ability to invade dsDNA polypurine-polypyrimidine duplexes, we attribute the single strand in the Y-strand to formation of a PNA_2_-DNA triplex-invasion complex (Fig 6B, structure 3). This result is in full accordance with the data obtained for the pMP141(9 repeats) as shown in Fig 4 and Fig 5, although it cannot, on its own, explain the pronounced BQQ-OP cleavage in the presence of this PNA. Thus, a triplex-invasion complex of the dsDNA may co-exist with H-DNA under these conditions (Fig 6B, structure 2). CTT-PNA may also bind to the GAA repeat forming a traditional PNA-DNA_2_ triplex (Fig 6B, structure 4) analogous to that formed by CTT-DNA oligonucleotides. Such a triplex is cleaved by BQQ-OP (Fig 2, lanes 2 and 5), but much less efficiently than pure DNA-triplexes, and thus could not account for the increased BQQ-OP sensitivity. Therefore the present data do not allow for a detailed quantitative structural description of the interaction of CTT-PNA with medium GAA repeats, but a combination of PNA triplex invasion as well as H-DNA (like) structures must be involved. The difficulties in interpreting this data may also reflect different kinetics of formation of the different DNA structures (B-DNA, H-DNA and triplex-invasion structures), which can transiently co-exist and hence consequently be detected by chloroacetaldehyde or BQQ-OP reactions.

### LNA Binding to H-DNA Forming FRDA (GAA)_115_ Repeats

**Fig 7:**
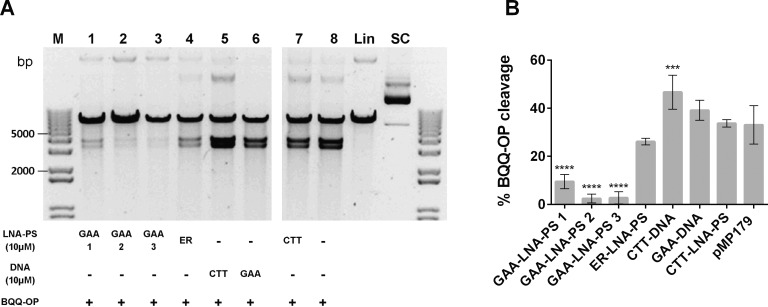
BQQ-OP mediated DNA cleavage of H-DNA forming (GAA)_115_ repeats in the presence of LNA-PS. **A)** Representative gel from pMP179(115 repeats) incubated with 10 μM LNA-PS (GAA-LNA-PS 1, 2 or 3 or ER-LNA-PS or CTT-LNA-PS, lane 1, 2, 3, 4 or 7, respectively), in the absence of oligonucleotide (lane 8) or with 10 μM ssDNA (CTT-DNA or GAA-DNA, lane 5 or 6, respectively) in buffer (10 mM sodium cacodylate, 100 mM NaCl, 2 mM MgCl_2_, pH 7.5). Triplex-specific cleavage was carried out in the presence of Cu^2+^ and MPA, and the plasmid was subsequently digested using ApaI. The two obtained DNA fragments have approximately 3814 and 3178 bp. Reference supercoiled (SC) and linearized (Lin) pMP179(115 repeats) and a molecular weight DNA ladder (M) are also shown. **B)** Graph represents the percentage of BQQ-OP mediated cleavage for pMP179(115 repeats) in the absence (indicate in the graph as pMP179) or in the presence of LNA- or DNA oligomers. Values indicate the ratio between the intensity of DNA double strand cleavage to the total band intensity from the respective lane and are expressed as mean±S.D. (n=3). *** *P*≤0.001, **** *P*<0.0001 in relation to plasmid in the absence of oligonucleotide (pMP179).

To examine whether another type of modified oligomer, with comparable dsDNA invasion properties as PNAs, could mediate results similar to those previously observed, the BQQ-OP cleavage assay was performed in the presence of CTT- or GAA-LNA-PS oligomers. Fig 7A shows, in an analogous way as in Fig 3B, that indeed in the presence of a 9-mer GAA-LNA-PS (lane 1) or 12-mer GAA-LNA-PS (lane 2 and 3) the BQQ-OP cleavage was almost abolished, presumably due to the destabilization of the H-DNA structure. When compared with the plasmid alone, a statistic significance (*P*≤0.0001) was determined for all GAA-LNA-PS ONs, however different mean±S.D. percentages of BQQ-OP cleavage were found depending of the ONs size, decreasing with increase of length (GAA-LNA-PS 1: 9.5±2.9%, while GAA-LNA-PS 2 and 3: 2.4±1.8% and 2.7±2.5%, respectively) (Fig 7B). Interestingly, is the lack of significance for the ER-LNA-PS ON (26.1±1.4%), showing that indeed the ON effect is sequence specific. Regarding CTT-LNA-PS, no significant increase in the BQQ-OP cleavage percentage was found (33.7±1.5%). These results support the hypothesis that modified GAA ONs can be used to destabilize triplex structures formed at (GAA)_n_ repeats.

### Monitoring the Effect of LNA Binding to (GAA)_115_ Containing Plasmids Using AFM

DNA topology is affected by formation of higher order DNA structures not the least at expanded triplet repeats. Atomic force microscopy (AFM) has been used to examine cruciforms, H-DNA and higher order structures at CAG and GAA repeats, respectively. In a similar way, we reasoned that oligomer binding to structure forming GAA expansions should be reflected by changes in DNA topology (and thereby morphology of circular DNA) and could be detectable by AFM. To examine this hypothesis, we monitored the effects of LNA binding to pMP179(115 repeats) using CTT-, GAA- or a scrambled-LNA (Table 1) and the binding conditions described above. We assumed that physiological buffers, containing Mg^2+^ and Na^+^, which are both required for DNA structure stabilization, would be sufficient for visualization of DNA [66,67]. However, while Mg^2+^ binds very weakly to mica surfaces, the presence of Na^+^ promotes the release of DNA molecules from the surface. This can be overcome by the addition of Ni^2+^ ions to the buffer. Thus, the Ni^2+^ concentration used in this work permits suitable attachment of DNA to the mica surface, avoiding Ni^2+^ precipitation and poor DNA binding, occurring at higher and lower NiCl_2_ concentrations, respectively [60,68]. Studies by Billingsley et al. (2010) demonstrate that the hydration of the sample during AFM measurements is crucial to determine morphology of supercoiled plasmids. Specifically, they show that supercoiled samples prepared with Ni^2+^ with high hydration are more condensed, with a large number of crossovers, local conformation changes and more turns effects that seem to be topologically driven. They also postulate that under these conditions, the configuration is closer to the 3D solution form [69]. DNA supercoiling is also dependent on the plasmid integrity, and PeakForce^®^ tapping (PTM) mode, where the applied force can be controlled and decreased below the nano-Newton range, is a suitable technique for this type of measurements where the preservation of the sample is crucial [61].

**Fig 8:**
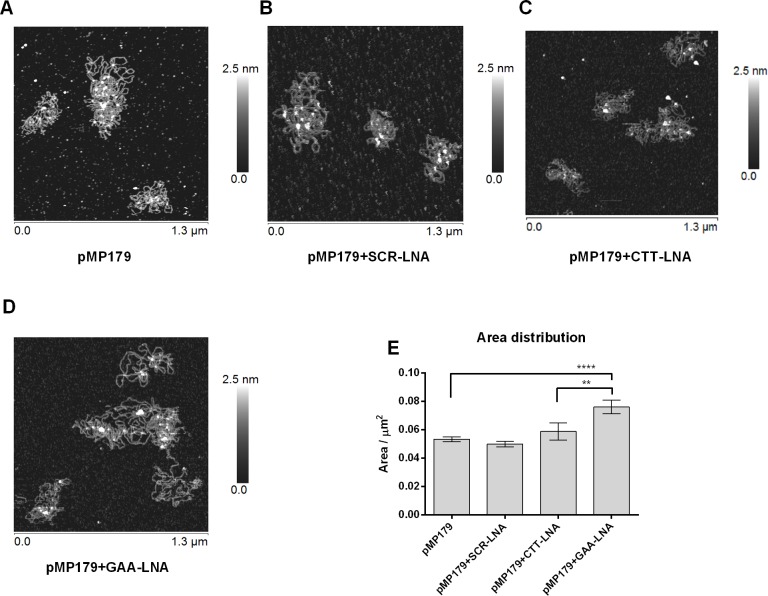
Topography analysis of pMP179 (115 repeats) in the absence or presence of LNA-PS ONs. Representative AFM topography images of 12.5 ng of supercoiled pMP179(115 repeats): **A)** in the absence of LNA; **B)** in the presence of SCR-LNA-PS oligomer; **C)** in the presence of CTT-LNA-PS or **D)** in the presence of GAA-LNA-PS 3. Sample preparation in a 10 mM HEPES supplemented with 5 mM Ni^2+^ solution, pH 7.4 was deposited onto fresh clean mica. Colour scales: 2.5 nm. Images were acquired in liquid with the Dimension FastScan, Nanoscope V operating in Peakforce^®^ tapping mode. **E)** Plasmid area distribution from different AFM overview fields of pMP179(115 repeats) supercoiled alone or in the presence of the different LNA ONs. The graph shows the mean±S.E.M. determined for each condition (pMP179 n=312; pMP179+SCR-LNA-PS n=185; pMP179+CTT-LNA-PS n=63; and pMP179+GAA-LNA-PS 3 n=216). A highly significant increase in the area occupied by the plasmids is observed in the presence of the GAA-LNA-PS 3, representing a more relaxed morphology. ** *P*≤0.01, **** *P*≤0.0001 in relation to plasmid in the absence of oligonucleotide (pMP179).

In view of these considerations, we performed AFM measurements by PTM in liquid in the presence of Ni^2+^ ions using pMP179(115 repeats) incubated in the presence or absence of LNA ONs. All analyses were performed under the same conditions. As shown in Fig 8, pMP179(115 repeats) the supercoiled state (Fig 8A) presents a very condensed morphology, resembling a compact sphere structure and this morphology is maintained in the presence of scrambled-LNA (Fig 8B) or CTT-LNA (Fig 8C) oligomers. However, in the presence of GAA-LNA (Fig 8D) a clearly more relaxed morphology was observed. As control, we performed similar AFM analysis using the parent plasmid pSPL3, lacking the GAA repeat sequence, in the absence or presence of the same set of oligomers, and in all cases we did not observe any change of the plasmid morphology (S1 Fig). This confirms that oligomer mediated change of plasmid structure is dependent on the presence of the GAA expansions.

To provide a quantitative analysis of the oligomer binding effect, detected by AFM, we measured the distribution (mean±S.E.M. values) of all the areas determined under each condition tested as shown in the graph of Fig 8E. On average, pMP179(115 repeats) occupies an area of 0.053±0.0016 μm^2^ (pMP179 SCR-LNA-PS: 0.050±0.0019 μm^2^ and pMP179 CTT-LNA-PS 0.059±0.0061 μm^2^) while in the presence of GAA-LNA-PS 3 an area of 0.076±0.0047 μm^2^ is observed, significantly increasing the area by 43.4%. Statistical analysis shows that GAA-LNA-PS 3 binding of the repeat-containing plasmid clearly has a significant effect (*P*≤0.0001), whereas CTT-LNA-PS shows no statistically significant difference as compared to the control (*P=*0.3740), consistent with the BQQ-OP cleavage data (Fig 7). In other words, GAA-LNA-PS 3 significantly changed the conformation of the GAA-repeat plasmid, both as compared to the control plasmid, and as compared to the CTT-LNA-PS treated plasmid (*P=*0.0071).

These results support the conclusion that GAA-LNA (but not CTT-LNA) in analogy to GAA-PNA can resolve triplex containing higher order structures thereby promoting a more relaxed structure that is topologically similar to duplex DNA.

## Conclusions

Chemical and structural probing of GAA repeats provides evidence for pyrimidine triplex (H-DNA) formation and the presence of different structures at the pathological repeats. Furthermore, we find that PNA and LNA sequence-specific targeting of Friedreich’s ataxia GAA repeat expansions can alter and resolve higher order DNA structures. BQQ-OP mediated triplex-specific cleavage of double strand DNA and chloroacetaldehyde chemical modification of single strand DNA regions at (GAA)_n_ repeats demonstrate that GAA-PNA binding result in a duplex invasion complex, that completely dissociates all detectable triplex containing higher order structures at this site, whereas this is not the case for CTT-PNA. Additionally, we obtained a similar pattern using LNA based ONs. Furthermore, a significant change in plasmid morphology in the presence of GAA-LNA was detected using atomic force microscopy. Our results suggest that DNA targeting by modified GAA-oligomers at expanded (GAA)_n_ repeats can be employed to examine the possible role of non-canonical DNA structures in *FXN* gene silencing and potentially applied to develop new nucleic acids-based therapeutic strategies in Friedreich’s ataxia disease.

## Supporting Information

S1 Dataset BQQ-OP cleavage raw data and statistical analysis. File contains all the data analysis regarding the BQQ-OP cleavage experiments in the presence or absence of or PNA or LNA oligomers.
(XLSX)

S2 Dataset AFM raw data and statistical analysis. File contains all the data analysis regarding the AFM experiments with pMP179 or pSPL3 in the presence or absence of LNA oligomers.
(XLSX)

S1 Fig Topography analysis of pSPL3 in the absence or presence of LNA-PS ONs. Representative AFM topography images of 12.5 ng of supercoiled pSPL3: A) in the absence of LNA; B) in the presence of SCR-LNA-PS oligomer; C) in the presence of CTT-LNA-PS or; D) in the presence of GAA-LNA-PS 3. Sample preparation in a 10 mM HEPES supplemented with 5 mM Ni^2+^ solution, pH 7.4 was deposited onto fresh clean mica. Colour scales: 2.0 nm. Images were acquired in liquid with the Dimension FastScan, Nanoscope V operating in Peakforce® tapping mode. E) Plasmid area distribution from different AFM overview fields of pSPL3 supercoiled alone or in the presence of the different LNA ONs. The graph shows the mean ± S.E.M. determined for each condition (pSPL3 n=159; pSPL3+SCR-LNA-PS n=152; pSPL3+CTT-LNAPS n=127; and pSPL3+GAA-LNA-PS 3 n=154). No statistic significance was found among all the treatments when compared to plasmid (pSPL3) in the absence of oligonucleotide.
(TIF)
