# Supplementary figures and images for: Disruption of Higher Order DNA Structures in Friedreich’s Ataxia (GAA)n Repeats by PNA or LNA Targeting
Source: PLoS One. 2016 Nov 15;11(11):e0165788. doi: 10.1371/journal.pone.0165788 (PMC5112992; doi:10.1371/journal.pone.0165788)

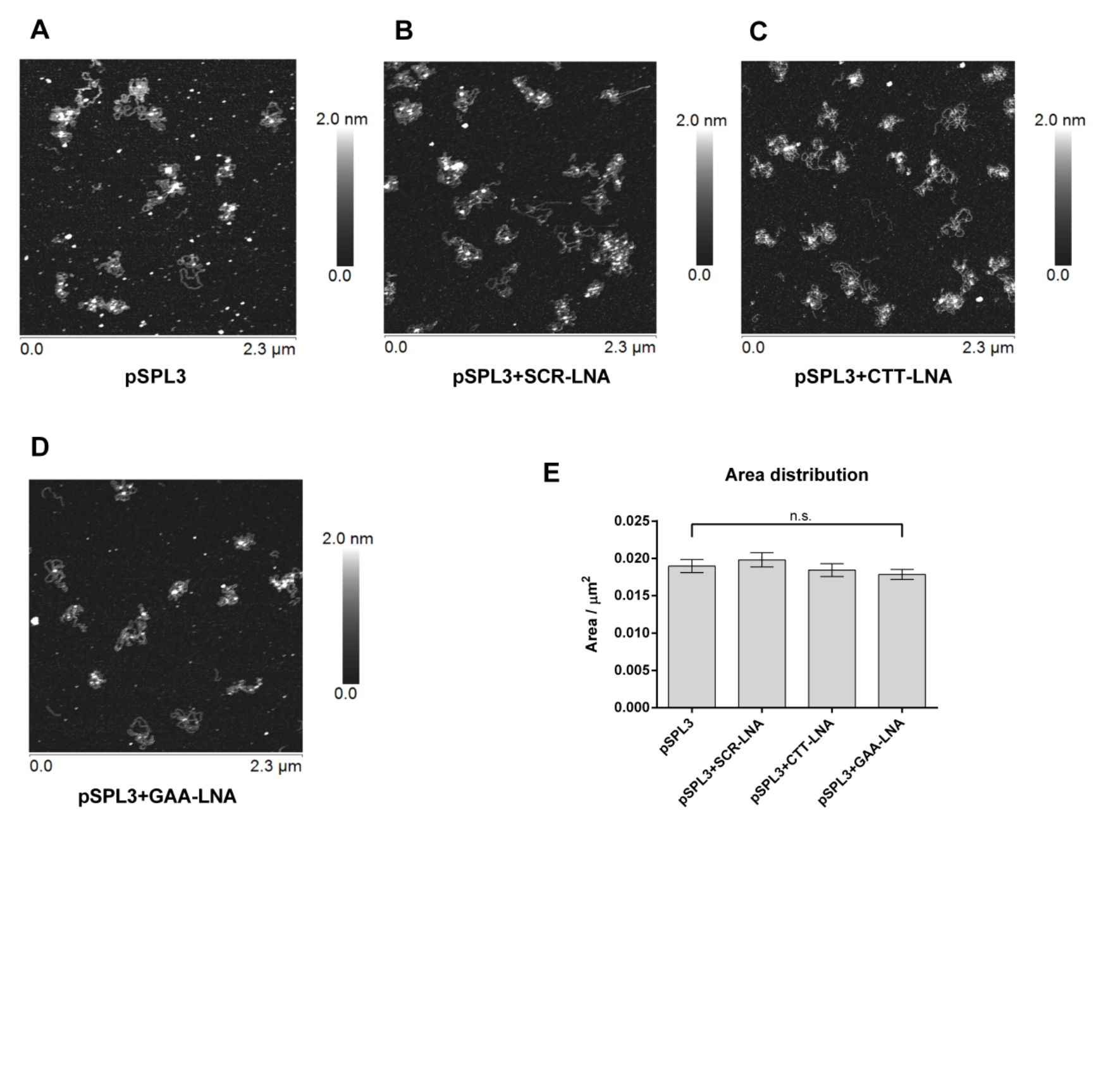

Supplement: S1 Fig — Representative AFM topography images of 12.5 ng of supercoiled pSPL3: A) in the absence of LNA; B) in the presence of SCR-LNA-PS oligomer; C) in the presence of CTT-LNA-PS or; D) in the presence of GAA-LNA-PS 3. Sample preparation in a 10 mM HEPES supplemented with 5 mM Ni2+ solution, pH 7.4 was deposited onto fresh clean mica. Colour scales: 2.0 nm. Images were acquired in liquid with the Dimension FastScan, Nanoscope V operating in Peakforce® tapping mode. E) Plasmid area distribution from different AFM overview fields of pSPL3 supercoiled alone or in the presence of the different LNA ONs. The graph shows the mean ± S.E.M. determined for each condition (pSPL3 n=159; pSPL3+SCR-LNA-PS n=152; pSPL3+CTT-LNAPS n=127; and pSPL3+GAA-LNA-PS 3 n=154). No statistic significance was found among all the treatments when compared to plasmid (pSPL3) in the absence of oligonucleotide. (TIF) [file pone.0165788.s003.tif]
